# Comparison of Hip and Knee Biomechanics during Sidestep Cutting in Male Basketball Athletes with and without Anterior Cruciate Ligament Reconstruction

**DOI:** 10.5114/jhk/162965

**Published:** 2023-07-15

**Authors:** Pinyada Warathanagasame, Prasert Sakulsriprasert, Komsak Sinsurin, Jim Richards, Jamie S. McPhee

**Affiliations:** 1 Biomechanics and Sports, Faculty of Physical Therapy, Mahidol University, Nakhon Pathom, Thailand.; 2 Allied Health Research Unit, University of Central Lancashire, Lancashire, United Kingdom.; 3 Department of Sport and Exercise Sciences, Manchester Metropolitan University Institute of Sport, Manchester, United Kingdom.

**Keywords:** rehabilitation, motor control, instability, injury

## Abstract

This study aimed to compare hip and knee biomechanics during sidestep cutting on the operated and non-operated sides in individuals with anterior cruciate ligament reconstruction (ACLR), and in an uninjured control group. Twenty male basketball athletes, 10 individuals with ACLR and 10 controls, were recruited. Hip and knee joint angles and angular velocities were investigated with a three-dimensional motion analysis system, and ground reaction forces (GRF) along with moments were collected during the deceleration phase of the stance limb during sidestep cutting maneuvers. We found significantly higher peak hip flexion, hip internal rotation angular velocities, and peak thigh angular velocity in the sagittal plane in the ACLR group. In addition, the peak vertical GRF and peak posterior GRF of the ACLR group were significantly higher than those of the control group. Univariate analyses indicated that the posterior GRF of the non-operated side was significantly higher than in the matched operated side in the control group. The operated and non-operated sides in male basketball athletes with ACLR showed alterations in hip and knee biomechanics compared with a control group, especially in the sagittal plane. Therefore, the emphasis of neuromuscular control training for the hip and the knee in basketball players with ACLR is required.

## Introduction

Anterior cruciate ligament (ACL) injuries are common in athletes, especially in those who regularly perform high-impact rotational activities such as basketball (Jamkrajang et al., 2022; Montalvo et al., 2019). The average annual rate of ACL injury in male basketball athletes was 0.08/1,000 athlete-exposure (Agel et al., 2016). ACL reconstruction (ACLR) is the standard management of ACL injury, and seventy-five percent of individuals with ACL injury undergo ACLR to restore knee function and allow return to sports at the pre-injury level (Sanders et al., 2016; Smith et al., 2014). However, previous studies have reported a high incidence rate of ACL re-injury after ACLR and returning to sports, with six times more injuries than healthy athletes, and a 33.3% re-injury rate on the operated side and 66.7% more ACL injuries on the non-operated side (Paterno et al., 2012, 2014; Schilaty et al., 2017).

The high incidence rate of ACL injuries has been reported to be related to alterations in the knee and hip biomechanics after ACLR on both the operated and non-operated sides, especially during the landing phase (Johnston et al., 2018; Lepley and Kuenze, 2018; Pratt and Sigward, 2017). Individuals with ACLR who presented a decrease in hip external rotator moment and asymmetry of knee extensor moment had a high incidence rate of ACL injury after ACLR (Johnston et al., 2018; Paterno et al., 2010). Nevertheless, the decreased knee flexion angle and knee flexion angular velocity represented more stiffness at the knee joint during the deceleration phase of landing. It affected increased vertical GRF related to increased anterior shear force at the knee and ACL loading (Yeow et al., 2009).

Athletes after ACLR exhibit impaired joint proprioception in the early phase of rehabilitation and at return to sports (Fremerey et al., 2000), which is associated with the alteration of corticospinal excitability (Rush et al., 2021). In addition, corresponding alterations of motor thresholds (MT) and motor evoked potentials (MEP) of the quadriceps have been reported on both sides after ACLR during the early stage (less than 24 months) and late stage (more than 24 months) of recovery (Rush et al., 2021). This could be explained by the weakness in the quadriceps as shown by a decrease in the single-hop distance on both sides compared with a healthy group (Chung et al., 2015; Laudner et al., 2015). This has been attributed to the alterations in neuromuscular control on both sides during dynamic movement in athletes with ACLR during the return to sports phase.

Neuromuscular control changes have been reported to affect the alteration of muscular activity and segmental movement during dynamic tasks (van Leeuwen, 1999). Previous studies showed the reduction of peak knee extensor moments on the operated side, and increases in the hamstring activation on the operated side during drop jump landings (Johnston et al., 2018; Pratt and Sigward, 2017; Smeets et al., 2020). In addition, the reduced hip extensor moment and reduced hip external rotator moment on the operated side during drop jump landings were reported (Johnston et al., 2018; Lepley and Kuenze, 2018). The reduced knee extensor moment and reduced hip external rotator moment were associated with the risk of secondary ACL injury after ACLR (Paterno et al., 2010). In addition, previous studies presented a correlation between the knee joint moment and knee, shank, and thigh angular velocities in sagittal and coronal planes (Dowling et al., 2012; Sigward et al., 2016). These represented the knee and segmental control of the operated side during movement, which may be related to the risk of secondary ACL injury after ACLR (Dowling et al., 2012).

Many studies on ACLR focused on the operated side, while the high incidence rate of secondary ACL injury was reported on both operated and non-operated sides (Dowling et al., 2012; Johnston et al., 2018; Lepley and Kuenze, 2018). In addition, the alterations of cortical excitability of quadriceps and isokinetic muscle strength on both operated and non-operated sides can affect the change of hip and knee joints biomechanics on both sides as well (Chung et al., 2015; Fremerey et al., 2000; Pratt and Sigward, 2017; Rush et al., 2021), which is particularly relevant in athletes who had a high risk of ACL injury and who performed dynamic and complex tasks with a high ACL injury risk such as sidestep cutting (Cochrane et al., 2007). Therefore, this study aimed to investigate hip and knee angles, hip, knee, shank, and thigh angular velocities, and hip and knee joint moments during sidestep cutting in male athletes with unilateral ACLR and compare them with those of healthy male athletes. We hypothesized that hip and knee angles, hip, knee, shank, and thigh angular velocities, and hip and knee joint moments during sidestep cutting in male athletes with ACLR would be different when compared to healthy male athletes.

## Methods

### Participants

Participants were recruited by convenience sampling by advertising through posters and social media, and were recruited from a professional league or a university basketball league. The inclusion criteria for the ACLR group were to have either a patellar tendon or a semitendinosus autograft surgical reconstruction, to participate in an ACLR rehabilitation program for at least 6 months before returning to sports activity at the same level as before injury, and to be more than 2 years after ACLR. The inclusion criteria for the control group were to be matched with the ACLR group regarding the level of competition and age, with no injury to the knee ligaments on both sides. Both legs were measured in the control group to be matched with the ACLR group according to the side of dominance. Participants were excluded if they had a history of lower extremity injury within 3 months preceding the study, a history of surgery in the lumbar and lower extremities, pain at the back or legs during movement, and a body mass index (BMI) greater than 30 kg/m^2^. This study was approved by the Central Institutional Review Board of the Mahidol University, numbered 2021/186.0709, and in accordance with the Declaration of Helsinki. All participants provided signed informed consent before data collection. G-power was used to calculate the sample size recruited from the peak shank angular velocity of the operated side versus the control group from pilot data, mean values were −423.682 and −492.288 ⁰/s (minus representing the flexion direction), standard deviation was 50.16 and 37.74 ⁰/s, respectively. In addition, a compensation for drop-out and missing data of 20% was considered, which yielded a sample of 10 in each group.

### Design and Procedures

This study had a cross-sectional design investigating hip and knee angles, as well as hip, knee, shank, and thigh angular velocities for all three planes of movement using a VICON motion analysis system with 10 infrared cameras (Oxford Metrics, UK), which was synchronized with force plates (AMTI, USA). Hip and knee joint moments were calculated using inverse dynamics with the calibrated anatomical system technique (CAST) (Cappozzo et al., 1995). Forty reflective markers were placed bilaterally on anatomical landmarks according to the lower-body CAST model, including the mid-point of the iliac crest, anterior superior iliac spine (ASIS), posterior superior iliac spine (PSIS), greater trochanter, medial and lateral femoral epicondyle, medial and lateral malleolus, posterior calcanei, distal head of the first and fifth metatarsals, proximal head of the fifth metatarsals, and cluster of four reflective markers were placed on the lateral side of thighs and shanks. The sampling rate was 200 Hz.

Demographic data and the knee and osteoarthritis outcome score (KOOS) (Chaipinyo, 2009), leg length from the anterior superior iliac spine to the medial malleolus, and strength assessment of the quadriceps and hamstring performed using a handheld dynamometer were recorded. In addition, the Lachman test, the posterior drawer test, as well as valgus, and varus stress tests were performed to screen the knee ligament (Rossi et al., 2011; Ostrowski, 2006). Leg dominance was determined by the side that the participant kicked a ball (Melick et al., 2017).

**Figure 1:**
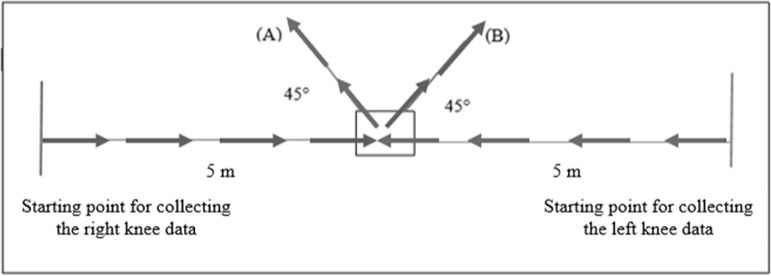
The laboratory setting with (A) indicating the cutting direction for the left knee, and (B) indicating the cutting direction for the right knee.

Participants then ran for 5 minutes with a self-selected speed to warm up, and then performed at least three practice sidestep cutting manoeuvres on each side to familiarise themselves with the testing procedures. The sidestep cutting manoeuvre consisted of a 5-m run before contact with the force plate on the floor and then changing the direction by 45° in the opposite direction to the tested limb which was indicated using a line on the floor (Figure 1). Biomechanical data were collected from five successful trials of sidestep cutting in the two directions which were randomized. Successful trials were defined by the participant’s foot landing within the area of the force plate, remaining in line with the direction of cutting, and performed at maximum speed (Pollard et al., 2015). A rest interval of 5 minutes was allowed between left and right side testing.

### Data Processing

**Figure 2:**
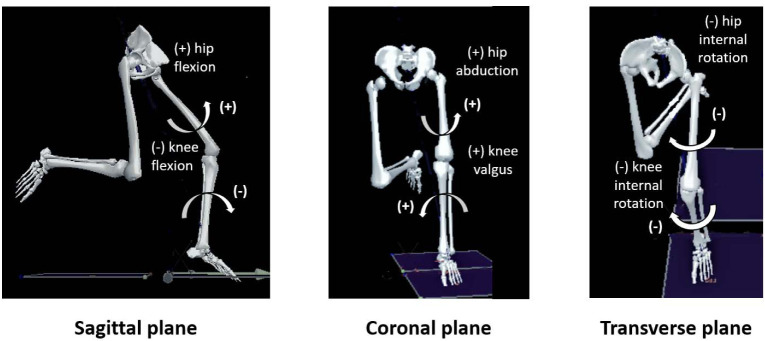
The definition of positive and negative values of knee, shank, and thigh angular velocities in the three planes.

Kinematic and kinetic data were imported into a visual 3D motion capture system (C-motion Inc., Germantown, USA) and filtered using 8 Hz and 50 Hz fourth-order zero-lag low-pass Butterworth filters, respectively. The changing direction phase was identified from the initial foot contact to toe-off using a threshold of 10 N of the vertical ground reaction force. The peak hip and knee angles, along with hip, knee, shank, and thigh angular velocities, ground reaction force (GRF), hip and knee joint moments which occurred within the initial 40% of the total stance phase (deceleration phase) were analysed (Figure 2).

### Statistical Analysis

Shapiro-Wilk tests were performed to check the normality of the data, and two-way ANOVA was carried out to compare differences of dependent variables between ACLR and control groups and between limbs. When data presented significantly different main effects or interaction, one-way ANOVA was used for post-hoc comparison of the operated and non-operated side in the ACLR group, and matched operated and non-operated side in the control group. All statistical analyses were performed using SPSS statistics version 23 (IBM, USA).

## Results

Twenty participants were recruited, 10 athletes two years after unilateral ACLR, and 10 healthy athletes. The mean age, BMI, quadriceps, and hamstring muscle strength were similar between the ACLR and the control group, however, the mean KOOS score of the ACLR group was lower than that of the control group, 85.53 and 95.64, respectively, where 0 represents extreme problems and 100 represents no problem (Table 1). These data confirm that the ACLR group had lower knee function than the control group.

**Table 1:** Participants’ characteristics.

Variables	*ACLR group*		*Control group*	
	Operated side	Non-operated side	Matched operated side	Matched non-operated side
Age (year) (Mean + SD)	30.80 + 5.61		30.20 + 5.85	
BMI (kg/m^2^) (Mean + SD)	24.67 + 1.48		25.41 + 2.82	
Reconstructed side (N, %) *-Left* *-Right*	7 (70%) 3 (30%)	N/A	N/A	N/A
Autograph type (N, %) *-Patellar tendon* *-Semitendinosus*	6 (60%) 4 (40%)	N/A	N/A	N/A
Dominant side (N, %) *-Left* *-Right*	1 (10%) 9 (90%)		1 (10%) 9 (90%)	
KOOS score (Mean + SD)	85.53 + 8.74		95.64 + 6.21	
Isometric quadriceps muscle strength (kg) (Mean + *SD)*	25.66 + 4.38	26.97 + 3.79	27.67 + 3.81	26.13 + 5.67
Isometric hamstring muscle strength (kg) (Mean + *SD)*	11.84 + 4.19	13.42 + 3.09	11.73 + 4.29	11.17 + 4.77
Running velocity at initial contact (m/s) (Mean + *SD)*	3.99 + 0.41	3.90 + 0.30	4.28 + 0.56	4.10 + 0.41

**Figure 3:**
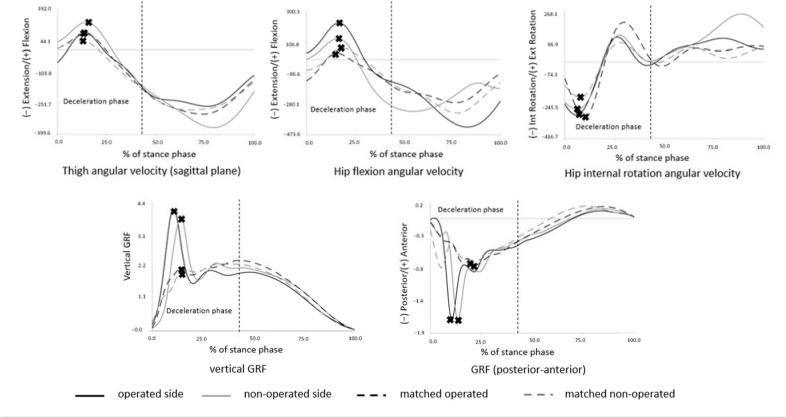
The graph pattern of kinematics (thigh angular velocity in the sagittal plane, hip flexion, and hip internal rotation angular velocities) and kinetics (GRF in posterior-anterior and vertical GRF). X marks were the values used for calculation.

The kinematic results presented significant main effects for a group in peak hip flexion angular velocity, peak hip internal rotation angular velocity, and peak thigh angular velocity in the sagittal plane (*p*-value = 0.009, 0.049, and 0.015, respectively). Post-hoc analysis revealed that peak thigh angular velocity in the sagittal plane of the non-operated side in the ACLR group was higher than of the matched non-operated side in the control group (*p*-value = 0.049). However, peak hip flexion and peak hip internal rotation angular velocities were not significantly different following univariate analysis (Table 2, Figure 3).

**Table 2:** Comparison of peak hip and knee angles, peak hip, knee and segmental angular velocities on main effects of between ACLR and control groups, and between sides.

Plane	Variables	ACLR group		Control group		Group effect F, *p*-value	Side effect F, *p*-value	Interaction effect F, *p*-value
		Operated side	Non-operated side	Matched operated side	Matched non-operated side			
**Sagittal**	Hip flexion angle (⁰)	50.35 + 6.09	51.09 + 6.60	52.04 + 6.70	51.95 + 7.98	0.345, 0.561	0.022, 0.883	0.037, 0.848
	Knee flexion angle (⁰)	−47.70 + 6.56	−50.08 + 4.20	−49.89 + 3.75	−49.93 + 6.71	0.350, 0.558	0.492, 0.487	0.459, 0.503
	Hip flexion angular velocity (⁰/s)	121.75 + 81.45	131.22 + 88.73	53.07 + 104.94	47.51 + 72.64	7.541, 0.009 *	0.005, 0.944	0.073, 0.788
	Knee flexion angular velocity (⁰/s)	−533.08 + 66.11	−575.56 + 72.76	−547.43 + 39.56	−536.88 + 70.82	0.364, 0.550	0.627, 0.434	0.173, 0.197
	Thigh angular velocity (⁰/s)	103.60 + 61.73	136.02 + 58.75	82.29 + 59.76	65.20 + 45.05	6.477, 0.015 *	0.160, 0.692	1.992, 0.167
	Shank angular velocity (⁰/s)	−430.11 + 46.71	−434.83 + 35.99	−465.40 + 52.78	−456.69 + 43.31	4.014, 0.053	0.020, 0.890	0.221, 0.641
**Coronal**	Hip abduction angle (⁰)	11.72 + 6.16	10.82 + 6.66	8.66 + 5.80	7.12 + 5.63	3.082, 0.088	0.403, 0.529	0.028, 0.869
	Knee valgus angle (⁰)	2.30 + 2.98	2.26 + 3.28	4.49 + 3.56	4.10 + 3.59	3.596, 0.066	0.041, 0.840	0.028, 0.867
	Hip abduction angular velocity (⁰/s)	136.62 + 65.12	127.86 + 51.68	129.99 + 73.99	112.26 + 52.20	0.327, 0.571	0.464, 0.500	0.053, 0.819
	Knee valgus angular velocity (⁰/s)	74.52 + 50.06	53.37 + 59.93	67.50 + 35.09	57.91 + 32.29	0.007, 0.932	1.128, 0.295	0.160, 0.692
	Thigh angular velocity (⁰/s)	60.77 + 25.25	80.46 + 33.54	63.10 + 37.47	58.62 + 29.97	0.937, 0.340	0.569, 0.456	1.436, 0.239
	Shank angular velocity (⁰/s)	68.87 + 28.15	56.67 + 15.03	54.93 + 38.94	44.54 + 36.20	1.768, 0.192	1.327, 0.257	0.008, 0.927
**Transverse**	Hip internal rotation angle (⁰)	−8.18 + 9.65	−9.47 + 8.91	−10.82 + 10.01	−9.26 + 9.70	0.160, 0.692	0.002, 0.966	0.222, 0.640
	Knee internal rotation angle (⁰)	−3.16 + 3.11	−5.12 + 3.10	−5.62 + 3.30	−6.22 + 2.86	3.317, 0.077	1.710, 0.199	0.484, 0.491
	Hip internal rotation angular velocity (⁰/s)	−262.80 + 133.46	−243.19 + 98.99	−204.02 + 109.27	156.48 + 106.15	4.165, 0.049 *	0.887, 0.352	0.153, 0.698
	Knee internal rotation angular velocity (⁰/s)	−88.91 + 40.88	−68.93 + 28.06	−80.23 + 33.35	−78.20 + 29.84	0.001, 0.978	1.086, 0.304	0.722, 0.401
	Thigh angular velocity (⁰/s)	−234.82 + 89.49	−213.01 + 86.34	−210.14 + 111.26	−178.63 + 114.63	0.851, 0.362	0.694, 0.410	0.023, 0.880
	Shank angular velocity (⁰/s)	−274.39 + 49.92	−257.33 + 52.35	−244.88 + 11.92	−245.64 + 89.93	0.656, 0.423	0.103, 0.750	0.123, 0.728

The values are reported as mean and standard deviation (SD)

* indicates significant difference (p-value < 0.05)

The kinetic results presented significant main effects for the group in peak vertical GRF and peak posterior GRF (*p*-value = 0.003 and 0.005, respectively). Post-hoc analysis revealed that peak vertical GRF of the non-operated side in the ACLR group was significantly higher compared to the matched operated side in the control group (*p*-value = 0.049). However, peak posterior GRF did not show a significant difference in univariate analysis (Table 3, Figure 3).

**Table 3:** Comparison of ground reaction force, hip, and knee joint moments between operated and non-operated sides in the ACLR group, and comparison between the ACLR and the control group.

Plane	Variables	ACLR group		Control group		Group effect F, *p*-value	Side effect F, *p*-value	Interaction effect F, *p*-value
		Operated side	Non-operated side	Matched operated side	Matched non-operated side			
	Vertical GRF (N/kg)	2.99 + 0.64	3.24 + 0.78	2.47 + 0.41	2.53 + 0.60	9.782, 0.003 *	0.615, 0.438	0.252, 0.618
**Sagittal**	Posterior GRF (N/kg)	−1.02 + 0.39	−1.12 + 0.40	−0.75 + 0.23	−0.74 + 0.32	8.838, 0.005 *	0.168, 0.684	0.257, 0.615
	Knee extensor moment (Nm/kg)	3.26 + 0.96	3.61 + 0.91	3.53 + 0.44	3.56 + 0.73	0.174, 0.679	0.573, 0454	0.406, 0.528
	Hip extensor moment (Nm/kg)	−4.14 + 1.01	−4.15 + 1.89	−3.70 + 1.10	−3.66 + 1.27	1.148, 0.291	0.001, 0.978	0.004, 0.951
**Coronal**	Medial GRF (N/kg)	0.77 + 0.28	0.85 + 0.28	0.72 + 0.18	0.71 + 0.18	1.622, 0.211	0.214, 0.646	0.329, 0.570
	Knee abductor moment (Nm/kg)	0.94 + 0.25	1.11 + 0.42	1.01 + 0.30	1.03 + 0.39	0.003, 0.958	0.756, 0.390	0.429, 0.516
	Hip abductor moment (Nm/kg)	1.98 + 0.33	2.12 + 0.62	2.07 + 0.28	2.12 + 0.32	0.109, 0.743	0.514, 0.478	0.082, 0.776
**Transverse**	Knee external rotator moment (Nm/kg)	0.18 + 0.07	0.21 + 0.11	0.26 + 0.08	0.22 + 0.07	3.670, 0.063	0.030, 0.864	1.429, 0.240
	Hip external rotator moment (Nm/kg)	0.10 + 0.11	0.16 + 0.14	0.13 + 0.13	0.11 + 0.13	0.043, 0.837	0.174, 0.679	0.785, 0.382

The values were reported mean and standard deviation (SD)

* indicates significant difference (*p*-value < 0.05)

## Discussion

This study aimed to compare hip and knee biomechanics including peak knee, shank, and thigh angular velocities, peak GRF, peak hip, and knee joint moments between male basketball athletes with ACLR who returned to sports and the control group on both operated and non-operated sides. The ACLR and control groups presented no difference in age, BMI, quadriceps and hamstring muscle strength, and running velocity. However, the KOOS score in the ACLR group was lower than in the control group, what reflected lower knee functions in the ACLR group.

Male basketball athletes with unilateral ACLR who returned to sports exhibited greater hip flexion, internal rotation angular velocities, and thigh angular velocity during knee flexion than healthy athletes, while the hip flexion and hip internal rotation angle were not different between the groups. It represented the ability of hip movement control that healthy basketball athletes could control the hip to move slower than basketball athletes with ACLR at a similar angle. The previous studies presented the greater movement of hip flexion and internal rotation during the deceleration phase of sidestep cutting and single-limb landing in athletes after ACLR and returned to sports (Lepley and Kuenze, 2018; Samaan et al., 2016). Especially, the non-operated side had the highest thigh angular velocity during knee flexion. This finding reflected the alteration of movement control on the non-operated side after ACLR too. The reduction of knee and shank angular velocities during the deceleration phase on both operated and non-operated sides was in accordance with the previous studies (Abd Razak et al., 2017; Lepley and Kuenze, 2018). Therefore, the results of this study indicate that more movement control at the knee constitutes protective strategies for reducing the knee loading, and compensating for greater movement of the hip joint.

Peak vertical GRF of the operated and non-operated sides in the ACLR group was greater than in the control group. This result is different from that of the study by Hughes et al. (2020) that presented reduced peak vertical ground reaction force on the operated side during double-limb landings. These divergent results may be due to the different tasks of the previous study which focused on the asymmetry between the ACLR and the non-operated side. In addition, the increased peak vertical GRF is related to the increased anterior shear-force of the knee, and is one of the factors that increase the risk of ACL injury (Pflum et al., 2004). Posterior GRF of the operated and non-operated sides were higher than in the control group, and correlated with increased knee anterior shear-force and shear-force in the frontal plane of the knee (Sell et al., 2007; Sigward et al., 2015; Yom et al., 2019). This is related to an increased risk of ACL injury on both sides after ACLR (Sigward et al., 2015). The knee and hip moments had no significant difference between the both sides and groups. However, the results of this study exhibited the pattern of an increased hip and a decreased knee extensor moment on the operated side during the deceleration phase of sidestep cutting, while the non-operated side and control legs presented similar hip and knee extensor moments. Considering a previous study which was a case study, the same pattern of the hip and knee joint moment during sidestep cutting was found in female soccer players who returned to sports (Cd et al., 2017).

Results of this study indicate that alterations of hip and knee biomechanics of both operated and non-operated sides may still be found after more than two years of ACLR which results in the changed neuromuscular control of hip and knee joints and thus, an increased ACL injury risk. Therefore, clinicians should review the protocol for maintaining neuromuscular control and focus more on the non-operated side. This concerns especially athletes who perform more dynamic movement and contact activities to minimize risk factors for secondary ACL injury or knee injury on both operated and non-operated sides. This study has some limitations which should be acknowledged. We only focused on the period of a full return to sports, but did not investigate the period of the initiation of sports return, i.e., 12–18 months after ACLR. Such a comparison between the period of the initiation of sports return and the period of a full return to sports after ACLR in athletes could provide more understanding regarding neuromuscular control of the knee during sidestep cutting. Also, this study investigated male basketball athletes, thus other sports and female athletes should be considered in future studies.

## Conclusions

This study presents the alterations of hip and knee biomechanics on both operated and non-operated sides in return to sports training after ACLR, especially movements in the sagittal plane during the deceleration phase of sidestep cutting. The results indicate the alteration of hip movement control on both operated and non-operated sides after ACLR. Additionally, vertical GRF and posterior GRF of both operated and non-operated sides were greater than in the control group.
